# Optimization of the Preparation Conditions of Borneol-Modified Ginkgolide Liposomes by Response Surface Methodology and Study of Their Blood Brain Barrier Permeability

**DOI:** 10.3390/molecules23020303

**Published:** 2018-01-31

**Authors:** Zhiyang Lv, Yuwei Yang, Jie Wang, Jing Chen, Junsong Li, Liuqing Di

**Affiliations:** 1 College of Pharmacy, Nanjing University of Chinese Medicine, Nanjing 210046, China; lvzhiyang83@163.com (Z.L.); pharm_chenjing@163.com (J.C.); guxm_2010@126.com (J.L.); 2 Hanlin College, Nanjing University of Chinese Medicine, Taizhou 225300, China; hlxynursing@163.com (Y.Y.); wangjie199084@126.com (J.W.); 3 Jiangsu Engineering Research Center for Efficient Delivery System of TCM, Nanjing 210046, China; 4 Nanjing Engineering Research Center for Industrialization of Chinese Medicine Pellets, Nanjing 210046, China

**Keywords:** ginkgolides, liposome, borneol, response surface methodology, blood-brain barrier permeability

## Abstract

Ginkgolides (GG), containing ginkgolide A (GA), ginkgolide B (GB) and ginkgolide C (GC), are mainly prescribed for ischemic stroke and cerebral infarction. However, the ginkgolides can hardly pass the blood-brain barrier (BBB) into the brain. The purpose of this study was to prepare borneol-modified ginkgolides liposomes (GGB-LPs) to study whether borneol could enhance the transport of ginkgolides across the BBB. The preparation conditions of GGB-LPs were optimized by a response surface-central composite design. Also, pharmacokinetics and biodistribution studies of GGB-LPs were conducted using UPLC-MS. The optimal preparation conditions for GGB-LP were as follows: ratio of lipid to drug (*w*/*w*) was 9:1, ratio of phospholipid to cholesterol (*w*/*w*) was 7:1, and hydrate volume was 17.5 mL. Under these conditions, the GGB-LP yield was 89.73 ± 3.45%. With GGB-LPs, borneol significantly promoted the transport of ginkgolide across the BBB. The pharmacokinetic parameters of GGB-LP were significantly improved too, with T_max_ of 15 min and a high drug concentration of 3.39 μg/g in brain. Additionally, the drug targeting index and relative uptake rate of GGB-LP was increased. Borneol-modified ginkgolide liposomes can thus potentially be used to improve the BBB permeability of gingkolide formulations.

## 1. Introduction

Ginkgolides (GG) are the major bioactive components found in the *Ginkgo biloba* leaf (*G. biloba*), mainly consisting of ginkgolide A (GA), ginkgolide B (GB), ginkgolide C (GC), etc. These compounds have been recognized as antagonists of platelet activating factor (PAF) receptor, and GB is considered to be the most powerful PAF antagonist among them [1,2,3]. GG possesses many pharmacological activities, including improving cerebral circulation, expanding coronary vessels, cardioprotective, inhibiting platelet aggregation, reducing vascular permeability and preventing atherosclerosis, and thus has been widely used in the treatment of neurological and cardiovascular diseases [4,5]. 

**Figure 1:**
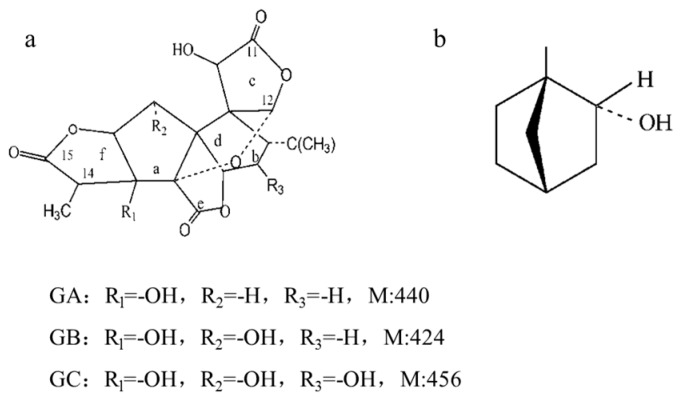
Chemical structure of ginkgolides (**a**) and borneol (**b**).

Ginkgolides (C_20_ compounds), belonging to the diterpene lactones, have a cage-like molecular structure (Figure 1a) with three lactone rings and one spirononane ring [1]. The structural differences between GA, GB and GC only depend on the number of hydroxy groups and their position. However, all of them share poor oral bioavailability related to their low solubility. After going through the systemic circulation, the lactone ring of ginkgolides is easily hydrolyzed, which remarkably reduces the bioavailability of ginkgolides. Because of their poor solubility and low bioavailability, the therapeutic effects of ginkgolides are greatly reduced [6,7]. During the last decades, pharmaceutical technologies such as microemulsions and solid dispersions have been tested and proved to improve the absorption of *G. biloba* flavonoids in studies of the oral absorption mechanism, pharmacokinetics and pharmacodynamics of *G. biloba* extract. However, few studies focusing on *G. biloba* lactones were reported, and the existing pharmaceutical technologies could hardly solve the fundamental problems associated with the poor oral bioavailability of *G. biloba* lactones. In recent years, *G. biloba* lactone injection has been widely used clinically in China. However, as expected, the amount of ginkgolides in the brain tissue was only 0.02% of the dosage at 10 min after injection, accompanied by uneven distribution of the ginkgolides in the brain. Because of the easily hydrolyzed lactone ring and rapid distribution to the stomach, duodenum, liver and other parts, few ginkgolides can be detected 30 min after injection. Therefore, the concentration of ginkgolides transported into the brain is the limiting step of *G. biloba* lactone against cerebral ischemia injury [8].

The blood-brain barrier (BBB) is an anatomical defense barrier between the brain and blood. Because of its extensive tight junctions and energy-dependent efflux transporters, BBB controls substance transport from the blood to the barrier, and plays an important role in protecting the central nervous system from toxic substances and serving to maintain brain homeostasis. The BBB protects the brain from unwanted substances, while, at the same time, limits the transport of many drugs into the brain. To date, the curative effect of drugs for the central nervous system is still greatly hampered by the existence of the BBB [9,10]. Recent pharmacological studies have shown that aromatic refreshing traditional Chinese medicines (TCMs), such as borneol, musk, styrax, benzoinum and Tatarinow Sweetflag Rhizome, can induce resuscitation and modify the permeability of BBB, promoting the entry of other drugs into the brain with brain protective effects. As a representative, borneol (Figure 1b) is a naturally occurring monoterpenoid compound in a class of ‘orifice-opening’ agents often used for resuscitative purposes in TCM. Borneol is divided into natural borneol (NB) and synthetic borneol (SB). SB consists of D-borneol and isoborneol, while NB contains only D-borneol [11,12,13,14,15]. A growing body of evidence confirms that borneol is able to loosen intercellular tight junctions, and thus open the BBB and enhance the distribution of drugs in the brain [16,17,18]. Therefore, borneol is believed to be an effective and promising adjuvant that can improve drug delivery to the brain.

Herein, we chose NB to modify *G. biloba* lactone liposomes. Moreover, we hypothesized that it may improve the BBB permeability of ginkgolides. Liposomes are synthetic membranes and vesicles that consist of various phospholipids and cholesterol, and have good affinity for cell membranes. Liposomes possess a phospholipid bilayer structure, and contribute to improving BBB permeability and increasing the stability of encapsulated drugs [19,20,21]. Due to their biocompatibility, biodegradability, low toxicity, hydrophobicity, hydrophilicity, and flexible structure, liposomes are known for their potential and actual uses in targeted drug delivery. However, ordinary liposomes are easily taken up by phagocytic cells and accumulate in the reticuloendothelial system (RES), which results in short residence times in the body [22,23]. Although some liposomes possess active targeting effects by attaching specific ligands (e.g., antibodies, peptides, nucleic acids, folic acid, sugars) [24,25,26,27], a saturation phenomenon may occur between specific ligands and target molecules [28]. 

In the present study, we prepared ginkgolide liposomes modified with borneol, and hypothesized that it might improve the BBB permeability of ginkgolides. The effective factors in the preparation of borneol-modified liposome (GGB-LP) were optimized by Response Surface methodology (RSM), and the blood-brain barrier permeability was compared between GGB-LP and ginkgolides liposomes without borneol (GG-LP). This work will provide a theoretical basis for further in vivo experiments to study whether GGB-LPs could promote the neuroprotective activity of *G. biloba* lactones against cerebral ischemia injury.

## 2. Results and Discussion

### 2.1. Preparation of GGB-LP

#### 2.1.1. Effect of Phospholipid/Drug Weight Ratio on Encapsulation Efficiency (EE) and Size

**Figure 2:**
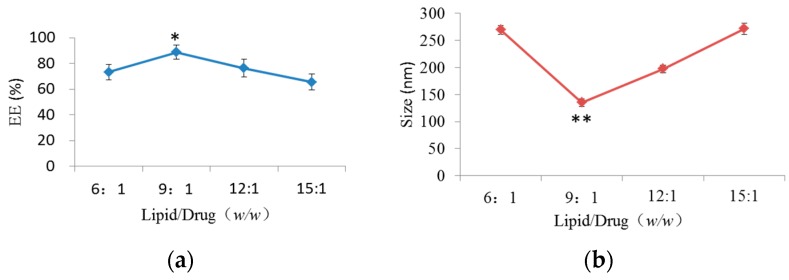

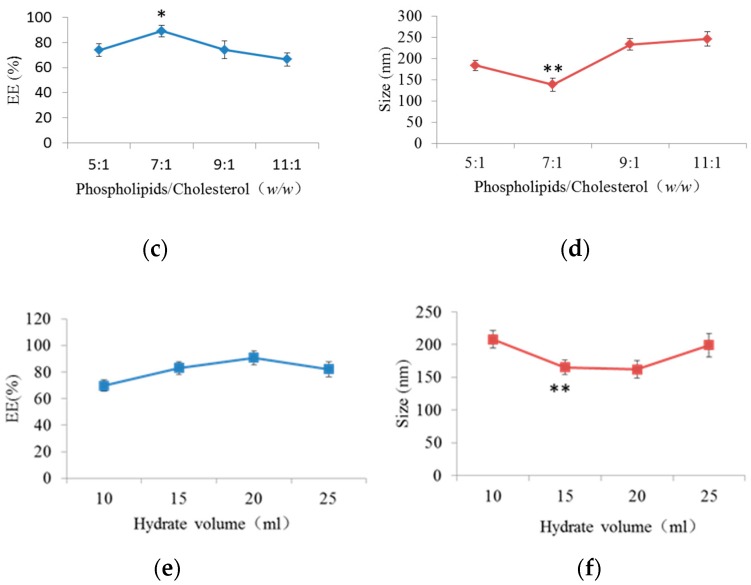
The influence of different single factors on the EE (**a**–**c**, %) and size (**d**–**f**, nm) of GGB-LP. * *p* < 0.05, ** *p* < 0.01, vs. lipid/drug ratio at 6:1, or phospholipid/cholesterol weight ratio at 5:1, or hydrate volume at 10 mL.

To study the influence of the lipid/drug weight ratio on EE and particle size of GGB-LP, different lipid/drug weight ratio (6:1, 9:1, 12:1, 15:1) was studied while the other preparation parameters were controlled at the same level. The effect of lipid/drug weight ratio on EE of GGB-LP is shown in Figure 2a. EE increased when the lipid/drug weight ratio ranged from 6:1 to 9:1. When the lipid/drug weight ratio was 9:1, EE of GGB-LP was highest (87.6%). Figure 2b shows that particle size of GGB-LPs was significantly reduced with the increase of lipid/drug weight ratio. When the lipid/drug weight ratio exceeded 9:1, the particle size increased. Therefore, the lipid/drug weight ratio of 9:1 was conducive to the smallest particle size.

#### 2.1.2. Effect of Phospholipid/Cholesterol Weight Ratio on EE and Size

Figure 2c,d present the effect of phospholipid/cholesterol weight ratio on GGB-LP preparation. Preparation experiments were carried out at different phospholipids/cholesterol weight ratios (5:1, 7:1, 9:1 and 11:1). It was found that the phospholipids/cholesterol weight ratio of 7:1 was a turning point which helps get the highest EE. The particle size of the liposomes firstly did not change significantly with the increased phospholipids/cholesterol weight ratio, but with the further increase of mass ratio, particle size was shown to increase sharply, and the optimum mass ratio for particle size was 7:1 (Figure 2d).

#### 2.1.3. Effect of Hydrate Volume on EE and Size

GGB-LP preparations were carried out using different volume of phosphate buffer pH 7.4 (1, 15, 20, 25 mL), while the other preparation conditions were kept unchanged. The influence of hydrate volume on EE and size was shown in Figure 2e,f. It was clear that the EE increased to reach its maximum when the volume of phosphate buffer pH 7.4 was 20 mL. Figure 2f shows that 20 mL of hydrate volume resulted in the lowest particle size.

### 2.2. Optimization of GGB-LP Preparation by RSM

Based on the results of single-factor experiments, 20 experimental runs were used for optimizing the three individual parameters in a central composite design (CCD), which were also used to maximize the EE (Y_1_, %) and minimize the particle size (Y_2_, nm) responses of the GGB-LP. By applying multiple regression analysis, the results of responses on three independent factors was shown in Table 1. The model was assessed using the multiple correlation coefficients (*R*^2^) and confidence interval (P) of the adjusted model. The values of regression coefficients were calculated, and the response variable and the test variables were related by the following second-order polynomial equation:

The model equation for EE is as follows:

$$\mathrm{EE}\;\left(\%\right)=88.18+1.78X_{1}-0.62X_{2}-0.11X_{3}-3.09X_{1}\;X_{2}+0.36X_{2\;}X_{3}+1.14X_{1\;}X_{3}\;-4.93X_{1\;}{}^{2}-5.53X_{2\;}{}^{2}-5.68X_{3\;}{}^{2}$$

The model equation for size is as follows:

$$\mathrm{Size}\;\left(\mathrm{nm}\right)=119.20-1.86X_{1}+24.50X_{2}-5.34X_{3}-11.61X_{1}\;X_{2}-8.70X_{2\;}X_{3}\;+9.57X_{1\;}X_{3}+67.58X_{1\;}{}^{2}+49.67X_{2\;}{}^{2}+32.93X_{3\;}{}^{2}$$

The statistical significance of the regression model was checked by *F*-test and *P* value, and the analysis of variance (ANOVA) for the response surface quadratic model was presented in Table 2 and Table 3. The determination coefficient (for EE, *R*^2^ = 0.9627; for size, *R*^2^ = 0.9823) showed by ANOVA of the quadratic regression model, indicating that the model was highly significant and adequate for prediction within the range of experimental variables. It can be seen that the lack of fit value of the mathematical models for EE and size were 0.58 and 0.25381 respectively, which was not significant relative to the pure error. Table 2 and Table 3 show that the independent variables (*X*_1_, *X*_2_ and *X*_3_) and the interaction terms (*X*_1_*X*_2_, *X*_2_*X*_3_, *X*_1_*X*_3_) significantly (*p* < 0.001) affected the EE and size of GGB-LP. The *R*^2^_Adj_ of 0.9291 in Table 2 and *R*^2^_Adj_ of 0.9663 in Table 3 indicated the good degree of fitting of the model.

**Figure 3:**
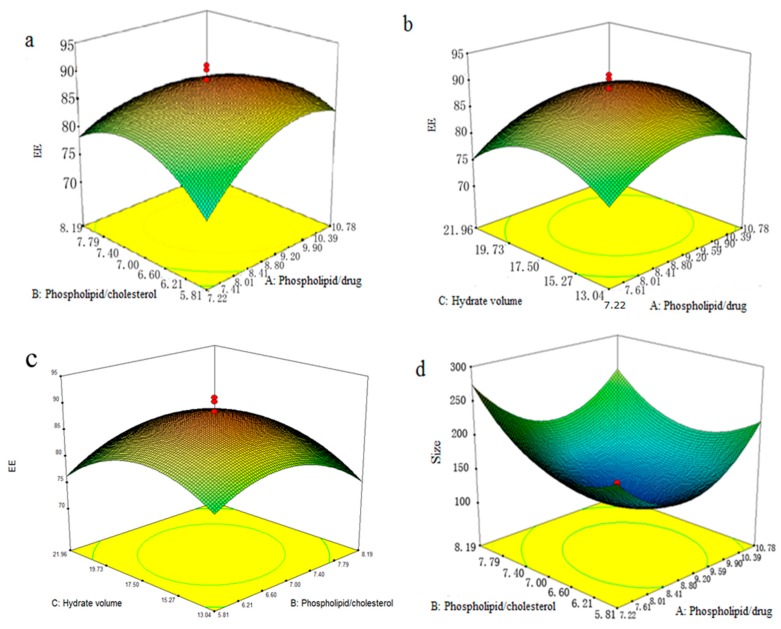

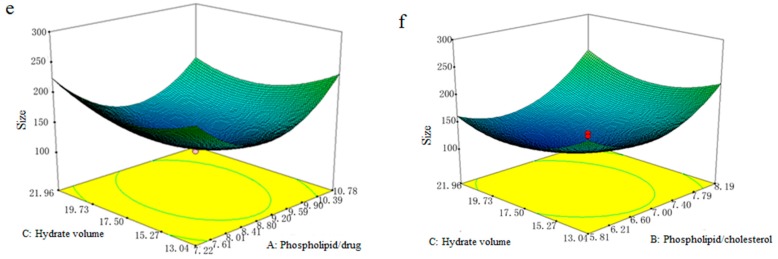
(**a**) 3D response surface plots for the influence of the phospholipid/drug ratio and phospholipid/cholesterol ratio on EE; (**b**) 3D response surface plots for the influence of the phospholipid/drug ratio and hydrate volume on EE; (**c**) 3D response surface plots for the influence of the phospholipid/cholesterol ratio and hydrate volume on EE; (**d**) 3D response surface plots for the influence of the phospholipid/drug ratio and phospholipid/cholesterol ratio on size; (**e**) 3D response surface plots for the influence of the phospholipid/drug ratio and hydrate volume on size; (**f**) 3D response surface plots for the influence of the phospholipid/cholesterol ratio and hydrate volume on size.

To show the type of interactions between the variables and the relationship between responses and experiment levels of each variable, the 3D response surface plots for EE and size of GGB-LP are profiled in Figure 3a–f.

Figure 3a depicts 3D response surface plots of the interaction effect of the phospholipid/drug ratio (*X*_1_) and phospholipid/cholesterol ratio (*X*_2_) on EE of GGB-LP. The EE increased at first, and then decreased after reaching to a certain degree. The peak value was the maximum EE point (91.13%) in these 3D plots.

The EE affected by the phospholipid/drug ratio and hydrate volume was seen in Figure 3b. With the increase of phospholipid/drug ratio, the EE initially increased, and then slightly reduced when the ratio was over 9:1 (*w*/*w*). The interaction effect between the two variables showed a positive effect on the response. 

The interaction effects of the phospholipid/cholesterol ratio and the hydrate volume (*X*_3_) were shown in Figure 3c. It was found that the EE initially increased with the increase of *X*_2_ and *X*_3_, but gradually decreased thereafter. EE reached a peak when the ratio of phospholipids to cholesterol was increased to 7:1 and the hydrate volume was 17.50 mL.

When the hydrate volume was fixed, the influences of phospholipid/drug ratio and phospholipid/cholesterol ratio on the size of GGB-LP were observed in Figure 3d. Phospholipid/drug ratio and phospholipid/cholesterol ratio had a detrimental impact on the size of GGB-LP. The size initially reduced with the increase of phospholipid/drug ratio and phospholipid/cholesterol ratio_._ However, the size increased when phospholipid/drug ratio and phospholipid/cholesterol ratio were over 9:1 and 7:1, respectively. 

The interaction influence of the phospholipid/drug ratio and hydrate volume was shown in Figure 3e. The result of response surface indicated that a minimum size (129.04 nm) was obtained when the phospholipid/drug ratio was 9:1, and the hydrate volume was 17.50 mL.

The selected variable conditions (phospholipid/cholesterol ratio and hydrate volume) also had impact on the size of GGB-LP. According to Figure 3f, when the phospholipid/cholesterol ratio and hydrate volume were 7:1 and 17.50, respectively, the minimum size point in these 3D plots was 129.04 nm.

### 2.3. Verification of the Predictive Model

**Figure 4:**
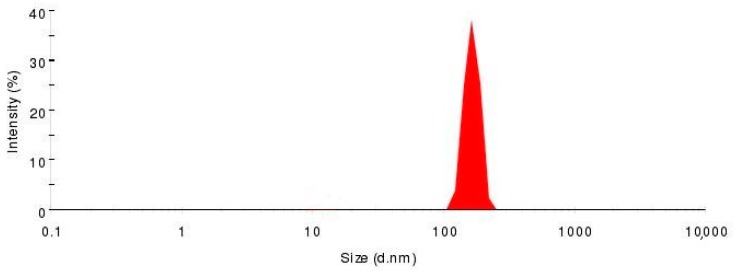
Intensity distribution of GGB-LP.

The optimized ranges of GGB-LP preparation conditions from 3D response surface plots were as follows: phospholipid/drug ratio (*X*_1_): 8.8:1–9.2:1; phospholipid to cholesterol ratio (*X*_2_): 6.8:1–7.2:1; hydrate volume (*X*_3_): 16.65–18.35 mL. To validate the adequacy of the model equation, three parallel experiments were conducted under the optimized conditions (phospholipid/drug ratio of 9:1, phospholipid to cholesterol ratio of 7:1, hydrate volume of 17.5 mL, and then responses were measured. The maximum predicted and experimental values of EE and particle size were given in Table 4. The actual EE of GGB-LP was 89.73 ± 3.45% (*n* = 3) and the mean size of GGB-LP was 128.01 ± 5.91 nm (*n* = 3). PDI was 0.142, indicating a relatively narrow particle size (Figure 4). The zeta potential was −27.2 mV, which showed that the GGB-LP would have a good dispersion. The predicted values of EE and size were 87.56% and 129.04 nm (Table 4). The deviations of EE and size between predicted values and experiment values were 2.47% and 0.79% respectively, which indicated that the model was adequate for the preparation process.

### 2.4. In Vitro Cell Uptake Study for Evaluation of BBB Penetration Potential of GGB-LP

**Figure 5:**
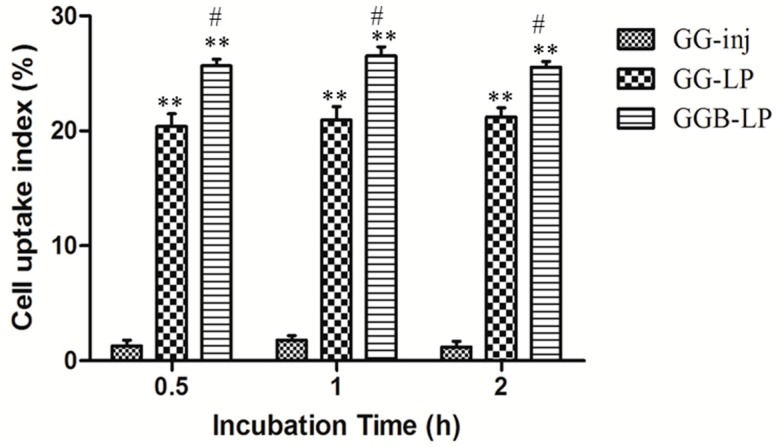
In vitro bEnd.3 cell uptakes of GGB-LP, GG-LP, and GG-inj during incubation at 37 °C (mean ± SD, *n* = 3), ** *p* < 0.001, vs. GG-inj; ^#^ *p* < 0.05, vs. GG-LP.

In this study, we used rat brain endothelia cells, bEnd.3, to mimic the endogenous microvascular endothelial cells, which has been reported to be a suitable BBB model for in vitro brain delivery studies. The in vitro cell uptake index of GGB-LP, GG-LP, and GG-inj was evaluated in bENd.3 cells, with incubation at 37 °C for 0.5, 1 and 2 h (Figure 5). The incubation time did not affect the uptake level in all three groups. However, GGB-LP showed significant higher uptake compared to GG-LP or GG-inj. The significant higher uptake values (*p* < 0.001) between GGB-LP (or GG-LP) and GG-inj in bEnd.3 cells indicated that the enhanced uptake efficacy was mediated by liposomes.

### 2.5. Brain Targeting and Biodistribution Studies of GGB-LP

In this study, three different formulations (GG-inj, GG-LP, GGB-LP) were administered i.v. to mice via the tail vein. According to the dosing regimen of marketed formulation, the administration concentrations of GB were 6.5 mg/kg for each group. The aim of this research was to investigate the influence of the addition of borneol in GGB-LP on the brain targeting and biodistribution of GB compared with GG-LPs. GG-inj was used as a control. The levels of GB in plasma and tissue samples were determined by UPLC-MS. The specificity, precision (RSD: 5.63%), stability (RSD: 3.28%), linear range of GB (0.01015–35.19 μg/mL) and extraction recovery of GB in plasma and tissue (90.18 ± 10.24%) of the UPLC-MS method met the testing requirements.

#### 2.5.1. Plasma Analysis

**Figure 6:**
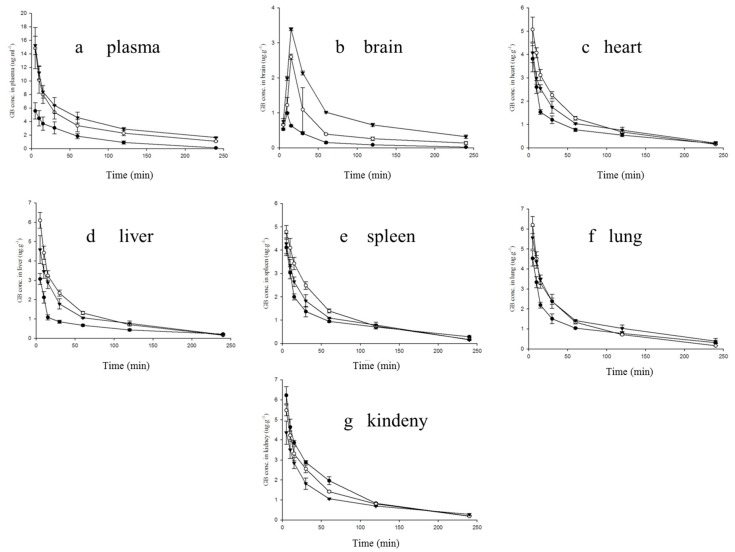
Mean GB concentration–time profiles in plasma (**a**); brain (**b**); heart (**c**); liver (**d**); spleen (**e**); lung (**f**) and kidney (**g**) after i.v. administration of GG-inj (●), GG-LP(○), GGB-LP(▼) to mice (*n* = 6, average ± SD).

The plasma concentrations of GB versus time are shown in Figure 6a. At 5 min after injection, the plasma concentrations of GB for GGB-LP and GG-LP were respectively 15.24 μg/mL and 14.84 μg/mL, while the value was only 5.56 μg/mL for GG-inj. For GG-inj, GB was almost undetectable at 240 min after the administration. However, the plasma drug concentration of GG-LP and GGB-LP groups were 10.31 and 15.58 times as high as GG-inj group respectively, and showed statistically significance (*p* < 0.05) compared with GG-inj control group. 

As shown in Table 5, the area under the curve (*AUC*_0__→__∞_) of GG-LP and GGB-LP were 991.58 and 1256.81 μg min mL^−1^ separately. They were 2.84 and 3.60 times as high as the *AUC*_0__→__∞_ of GG-inj, suggesting liposomes can significantly improve *AUC* of GB. Meanwhile, GG-LP and GGB-LP groups presented higher mean retention time (MRT) and lower plasma clearance rate than GG-inj, and GGB-LP had the highest MRT of 152.17 min and lowest plasma clearance rate of 0.0052. The increase of MRT may be related to the delayed release of liposomes in the blood and improved stability of GB by liposomes. 

#### 2.5.2. GB Distribution in Brain

The protective effect of ginkgolides injection on the brain largely depends on the GB concentration. The mean GB concentration–time curves of GG-inj, GG-LP and GGB-LP in brain are presented in Figure 6b. Primary pharmacokinetic parameters calculated by non-compartment model analysis are summarized in Table 5. As can be seen from Table 5, peak time (T_max_) of GB for GG-inj group was 10 min, while for GG-LP and GGB-LP groups the value were both 15 min. This may be resulted from the different way crossing through the blood-brain barrier of three formulations [29]. Liposomes permeate the BBB by the method of fusion and endocytosis, which may prolong the residence time in the capillaries. Compared with GG-inj, maximum concentration (C_max_), *AUC*_0__→__∞_ and MRT of GB for GG-LP and GGB-LP were higher, and the highest C_max_ (3.39 μg/mL), *AUC*_0__→__∞_ (272.12) and MRT (134.95 min) in the brain after administration were GGB-LP. The C_max_ of GB increased from 2.60 μg/mL for GG-LP to 3.39 μg/mL for GGB-LP with a corresponding increase in the *AUC*_0__→__∞_ from 136.85 μg min mL^−1^ for GG-LP to 272.12 μg min mL^−1^ for GGB-LP. *AUC*_0__→__∞_ of brain divided by the plasma drug concentration for GG-inj, GG-LP and GGB-LP groups were 11.91%, 13.80% and 21.65% respectively. These data suggested that borneol promoted GB distribution in the brain. The enhancement of GB level in the brain may be attributed to the enhancement of the blood-brain barrier permeability by borneol [30]. A large number of pharmacological and clinical literature have reported that borneol can enhance the amount of the drug delivered into the brain by increasing the content of 5-HT in the hypothalamus of animals to increase BBB permeability [5]. The DTI of GG-LP and GGB-LP were 1.15 and 1.82 respectively, which indicated that both of them were highly selective to the targeted brain regions. Similarly, the r_e_ of GB in GGB-LP group was higher than that in GG-LP. These results proved that ginkgolide liposomes modified by borneol could increase the blood brain barrier permeability of GB.

#### 2.5.3. GB Distribution in Other Tissues

Compared with GG-inj, GG-LP and GGB-LP can significantly change the tissue distribution of GB in mice, both of which exhibited the distinct characteristics different from GG-inj, and had a similar tissue distribution mutually. GG-inj had a high *AUC*_0__→__∞_ of 368.58 μg min mL^−1^ in kidney, with the C_max_ of 6.23 μg/mL and MRT of 67.36 min in kidney. These may be caused by the renal excretion, the major and fast excretion pathway of GB. The *AUC* of GG-LP and GGB-LP in heart, liver, spleen and lung were higher than GG-inj, especially in liver. GGB-LP had the highest *AUC*_0__→__∞_ of 303.15 μg min mL^−1^ in liver. As shown in Table 5, the r_e_ of GG-LP in liver, hear, spleen, lung and kidney were 1.67, 1.39, 1.25, 1.07 and 0.87, respectively, while the r_e_ of GGB-LP in liver, lung, heart, spleen and kidney were 1.48, 1.34, 1.28, 1.08 and 0.78. It is clear that the r_e_ of GG-LP and GGB-LP in the kidney were less than 1, indicating no targeting. The C_max_ and clearance (CL) of GG-LP were higher than GGB-LP in lung, which lead to the highest *AUC* of GGB-LP in lung (387.97 μg min mL^−1^) among three formulations. These may be attributed to the feature of borneol “aromatic go channeling the medicine upstream”, meaning borneol has a strong divergent action, which would bring some drug to the lung. On the basis of these results, we confirm that liposomes modified with borneol can reduce the amount of GB in the tissues, and increase the content of GB in the brain.

Many studies have shown that borneol can improve central nervous system drug delivery by enhancing blood-brain barrier permeability. The enhancement of the BBB permeability is associated with the modulation of multiple ATP-binding cassette transporters (e.g., P-glycoprotein), tight junction proteins, and predominant enhancement of vasodilatory neurotransmitters. Up to now, several pharmaceutical forms of borneol have been developed for improving the kinetic profiles of co-administered drugs and enhancing their delivery to the brain. Borneol is a promising agent that deserves further development as a BBB permeation enhancer for CNS drug delivery [30]. The enhancement on the penetration of GB into the brain in this study is probably mediated by borneol.

## 3. Materials and Methods 

### 3.1. Materials

Natural borneol (99.2% purity) was purchased from Shanghai Yuanye Biotechnologies Co., Ltd. (Shanghai, China). Ginkgolides (50.69% GB, 47.56% GA, 0.92% GC) was purchased from Nanjing Zixi Biological Products Co., Ltd. (Nanjing, China). Soya lecithin was bought from Shanghai Advanced Viecle Technology Co., Ltd. (Shanghai, China). Cholesterol was purchased from Sinopharm Chemical Reagent Co., Ltd. (Shanghai, China). Reference GA was obtained from the Jiangsu Institute for Food and Drug Control (Nanjing, China). Reference GB was obtained from the National Institute for Food and Drug Control (Beijing, China). Standard ketoprofen was obtained from the National Institute for the Control of Pharmaceutical and Biological Products (Beijing, China). Ginkgolides Injection was manufactured by Jiangsu Kanion Pharmaceutical Co., Ltd. (Lianyungang, China). Other chemicals and reagents used were chromatographic or analytical grade.

### 3.2. Preparation of Ginkgolides Liposomes Modified by Borneol (GGB-LP)

Liposomes were prepared by the thin film ultrasonic dispersion method [31]. Briefly, 25 mg of ginkgolides, 15 mg of borneol, and appropriate amounts of soya lecithin and cholesterol were placed in 250 mL pear-shaped flask. Absolute ethanol (50 mL) was then added and the mixture was dissolved completely with the aid of ultrasound. The organic solvent was evaporated under a reduced pressure at 40 °C and 60 g by a rotary evaporator. The thin film was hydrated with PBS (pH 7.4) by rotary evaporation under reduced pressure. Then liposomes were formed by ultrasonication for 15 min and the filtration with 0.22 μm microporous membrane.

### 3.3. Characterization of GGB-LP

The particle size of distributions, mean diameter and polydispersity Index (PDI) of GGB-LP were measured by a nanoparticle size measuring instrument (Malvern Instruments Ltd., Malvern, UK). The encapsulation efficiency (EE) of GB was determined using Sephadex G-50 gel filtration chromatography and high performance liquid chromatography-evaporative light scattering detector (HPLC-ELSD) analysis. The operation procedure was that 0.5 mL of GGB-LP was eluted by addition of 15 mL PBS (pH 7.4) at the flow rate of 1 mL/min. The eluent solution was placed into the tube and 1% Triton and 0.1 mol/L hydrochloric acid were added for demulsification. After adequate mixing, the resulting solution was centrifuged at 6000× *g* for 10 min, and then analyzed using HPLC-ELSD method. GB was quantified based on a standard curve constructed right after the sample analysis. The total amount of GB in the suspension was determined by dissolving the sample in 1% Triton and 0.1 mol/L hydrochloric acid, followed by centrifugation in the same condition and HPLC-ELSD analysis. The EE of borneol was also determined. Briefly, GGB-LP (5 mL) was centrifuged at 6000× *g* for 30 min, and the suspension was then analyzed by gas chromatography (GC) for the content of free borneol. For determination of the total amount of borneol, liposome solution was dissolved in 1% Triton and 0.1 mol/L hydrochloric acid, centrifuged (6000× *g*, 10 min) and analyzed using GC.

The equation for EE of GB or borneol in GGB-LP is as follows:

(1) $$\mathrm{EE}\%=W_{\mathrm{in}}/W_{\mathrm{total}}\times 100$$

where *W*_in_ is the amount of GB or borneol entrapped, and *W*_total_ is the total amount of GB or borneol used in the preparation [32].

### 3.4. Optimization of GGB-LP Preparation by Response Surface-Central Composite Design

The preparation conditions of GGB-LP were optimized by response surface-central composite design in this study. Based on the single factor tests, the weight ratio of phospholipid and drug (*X*_1_), the weight ratio of phospholipid/cholesterol (*X*_2_) and the volume of PBS (pH 7.4) used for hydration (*X*_3_, mL), were identified as key factors responsible for EE and size, and thus selected to optimize the preparation conditions of GGB-LP. The selected factors were subjected to response surface methodology (RSM) with central composite design. The ranges of independent variables are as follows: the range of *X*_1_ is 6 to 12; the range of *X*_2_ is 5 to 9; the range of *X*_3_ is 10 to 25. The range and central point values of the three independent variables investigated are summarized in Table 6. The dependent variables Y_1_ and Y_2_ were EE and particle size respectively. Three independent variables at five levels (3^5^) were adopted for response surface-central composite design by using Design-Expert software (Version 8.05, Stat-Ease Inc., Minneapolis, MN, USA). The coefficient of correlation (*R*^2^) and analysis of variance (ANOVA) were applied to evaluate suitability of the model. The fitted polynomial equations were expressed in 3D response surfaces [33].

### 3.5. Blood-Brain Barrier Permeability of GGB-LP

#### 3.5.1. In Vitro Cell Uptake Study for Evaluation of BBB Penetration Potential

bEnd.3 cells, the immortalized mouse brain endothelial cell line, were cultured in 90% Dulbecco’s modified Eagle’s medium supplemented with 4 mM L-glutamine, 1.5 g/L sodium bicarbonate, 4.5 g/L glucose and 10% fetal bovine serum, at 37 °C in a humidified environment with 5% CO_2_. In in vitro cell uptake studies, bEnd.3 cells were seeded at 10^7^ cells/100 μL in an Eppendorf tube and incubated at 37 °C with GGB-LP, GG-LP, and GG-inj, respectively for 0.5, 1 and 2 h. At each incubation time points, cells were separated by centrifugation (1500× *g*, 10 min), washed with PBS followed by the addition of ultrapure water to prepare the total cell lysate. An aliquot of 100 μL of the supernatant was transferred to another Eppendorf tube. Then both of the tubes with the respective GB contents were measured by UPLC-MS (supposed to be A and B, respectively). The cell uptake index was calculated from the formula (A−B)/(A+B).

#### 3.5.2. Animals and Administration of GG Formulations

Kunming strain mice (SPF level, male, weighing within 20 ± 2 g) used for this study were obtained from Qinglongshan Animal Breeding Farms (Nanjing, China). Before the experiment, the mice were fasted for 12 h but allowed free access to water. All animal experiments were conducted in full compliance with the guide for the care and use of laboratory animals and approved by the animal and ethics review committee of Nanjing University of Chinese Medicine, China. Mice were randomly and equally assigned to three different groups of GG formulations (*n* = 35 for each group) including ginkgolides injection (GG-inj) group, ginkgolide liposomes without borneol (GG-LP) group and GGB-LP group. Animals were given the GG formulations by intravenous injection via the tail vein, at a single dose of 6.5 mg/kg GB in the formulations. At the indicated times (5, 15, 30, 60, 120 and 240 min) after administration, blood was collected from the retrobulbar venous plexus. Blood samples were centrifuged at 3000× *g* for 6 min. Plasma was separated and stored at −20 °C until use. In addition to blood, tissue samples, including heart, liver, spleen, lung, kidney, and brain, were also collected after cervical dislocation. The tissues ware washed with physiological saline, dried with filter paper, weighed and stored at −20 °C until further experiment.

#### 3.5.3. UPLC-MS Analysis of GB in Plasma and Tissues 

Thawed plasma samples (100 μL), 10 μL of internal standard (ketoprofen, 0.1 μg/mL) and 20 μL hydrochloric acid (1 mol/L) were placed into a centrifuge tube, adequately mixed by vortexing for 1 min and 1 mL of ethyl acetate was added. Subsequently, the mixture was vortex-mixed for 5 min and centrifuged at 3600× *g* for 6 min. 900 μL of supernatant was concentrated under reduced pressure, and the residue is redissolved in 100 μL of the mobile phase, vortexed for 5 min, and centrifuged (8400× *g*, 10 min). The resultant supernatant (80 μL) was analyzed by UPLC-MS. For analysis of GB tissues, samples of heart, liver, spleen, lung, kidney, and brain were respectively homogenized with 3 mL of normal saline in a tissue homogenizer, mixed with 20 μL internal standard (ketoprofen, 0.1 μg/mL ),vortex-mixed for 3 min and centrifuged at 3600× *g* for 10 min. After centrifugation, the upper organic layer was transferred into a tube and evaporated to dryness in a stream of nitrogen. Mobile phase (100 μL) was added into the tube for redissolution. Then the samples were vortex-mixed for 3 min and centrifuged at 7200× *g* for 5 min. The resultant supernatant was analyzed by UPLC-MS. 

The levels of GB in plasma and tissues were assayed by UPLC-MS (Xevo TQD, Waters, Waters Corporation, Milford, MA, USA). The chromatographic conditions were as follows: Acquity UPL HSS T3 column (100 mm × 2.1 mm, 1.8 μm); column temperature, 40 °C; mobile phase was composed of 0.4% formic acid and methanol (30:70, *v*/*v*); flow rate, 0.25 mL/min; injection volume, 5 μL. The mass spectrometer was equipped with electro spray ionization (ESI) source, and the UPLC-MS analysis was operated in the positive ionization mode. Quantification was carried out using the multiple reaction monitoring (MRM) mode. Other MS parameters were set as following: the ion source temperature, 150 °C; desolvation temperature, 400 °C; flow rate of desolvation gas, 1000 L/h.

### 3.6. Data Analysis

Non-compartmental analysis of the pharmacokinetic data was performed by the statistical moment method using the DAS 2.0 pharmacokinetic program (Chinese Pharmacological Society, China). The key parameters of pharmacokinetics (such as *AUC*_0__→__∞_, MRT, T_max_, C_max_ and CL) were achieved from DAS 2.0 pharmacokinetic program. The tissue targeting efficiency was evaluated using the relative uptake rate (r_e_) and the drug targeting index (DTI). The r_e_ is calculated as follows: $r_{e}=AUC_{x}\left(lp\right)/AUC_{x}\left(\mathrm{GG}-\mathrm{inj}\right)$ (“*AUC_x_*’’ is the *AUC* of tissue x, and ‘‘lp’’ is the GG-LP or GGB-LP). r_e_ > 1 represents that the test group has certain tissue targeting compared with the control group, and the higher Re indicates the better tissue targeting; r_e_ < 1 indicates no targeting. DTI was obtained by the following equation: $\mathrm{DTI}=AUC_{x}\left(lp\right)/AUC_{p}\left(lp\right)/AUC_{x}\left(\mathrm{GG}-\mathrm{inj}\right)/AUC_{p}\left(\mathrm{GG}-\mathrm{inj}\right)$ (“*AUC_p_*’’ is the *AUC* of plasma), and the value of DTI > 1 was considered as the targeting distribution. Statistical comparisons were performed by one-way ANOVA for multiple groups, and *p* < 0.05 was considered statistically significant.

## 4. Conclusions

In this study, GGB-LPs were prepared, and the preparation conditions were optimized by RSM. The optimal preparation conditions for GGB-LP was as follows: ratio of phospholipid to drug (*w*/*w*) 9:1, ratio of phospholipid to cholesterol (*w*/*w*) 7:1, and volume of PBS for hydration 17.5 mL. The RSM result was reliable and the EE of GGB-LP was 87.56%. Besides, the average particle size of GGB-LP was 129 nm, suggesting that liposomes were well-distributed. In addition, the blood-brain barrier permeability study showed GGB-LPs had better permeability and delivered a higher concentration of GB in the brain, compared with GG-inj and GG-LP. Moreover, GGB-LP produced low level of ginkgolides accumulated in non-pathological organs, and the elimination of drug in these organs was fast. This study demonstrates that borneol can promote the transport of ginkgolide across the BBB, which may be attributed to the enhancement of blood-brain barrier permeability by borneol. With the aid of borneol, the clinical efficacy of ginkgolides against ischemic stroke would be enhanced, which will play an important role in the treatment of central nervous system.

## Figures and Tables

**Table 1:** Scheme of CCD with the results of responses on three independent factors.

No.	Independent Variables ^a^			Dependent Variables ^b^	
	*X*_1_	*X*_2_	*X*_3_	Y_1_	Y_2_
1	9.00	7.00	17.50	91.13	129.06
2	10.78	8.19	21.96	70.64	279.57
3	10.78	5.81	21.96	76.11	222.36
4	10.78	8.19	13.04	69.97	266.68
5	9.00	7.00	17.50	87.71	131.65
6	9.00	7.00	10.00	70.55	234.63
7	9.00	7.00	17.50	90.36	123.33
8	7.22	5.81	21.96	64.57	241.97
9	9.00	7.00	17.50	84.56	103.69
10	9.00	5.00	17.50	73.98	220.45
11	9.00	9.00	17.50	71.62	306.27
12	6.00	7.00	17.50	72.29	307.67
13	7.22	5.81	13.04	69.87	232.53
14	7.22	8.19	13.04	72.21	297.93
15	7.22	8.19	21.96	72.31	318.17
16	9.00	7.00	17.50	88.56	124.88
17	10.78	5.81	13.04	79.14	275.19
18	9.00	7.00	25.00	74.19	197.38
19	12.00	7.00	17.50	76.69	320.37
20	9.00	7.00	17.50	86.69	101.36

^a^ Independent variables: *X*_1_, the ratio of phospholipid to drug (*w*/*w*); *X*_2_, the ratio of phospholipid to cholesterol (*w*/*w*); *X*_3_, Hydrate volume(mL); ^b^ Dependent variables: Y_1_, EE (%); Y_2_, Particile size (nm).

**Table 2:** ANOVA of the regression model for the response variables (EE) and independent variables (*X*_1_, *X*_2_, *X*_3_).

Source	Sum of Squares	Df	Mean Square	*F* Value	*p*-Value Prob. > *F*	
Model	1186.40	9	131.82	28.66	<0.0001	significant
*X*_1_	43.24	1	43.24	9.40	0.0119	
*X*_2_	5.33	1	5.33	1.16	0.3072	
*X*_3_	0.15	1	0.15	0.033	0.8596	
*X*_1_*X*_2_	76.38	1	76.38	16.60	0.0022	
*X*_1_*X*_3_	1.01	1	1.01	0.22	0.6497	
*X*_2_*X*_3_	10.35	1	10.35	2.25	0.1645	
*X*_1_^2^	350.73	1	350.73	76.24	<0.0001	
*X*_2_^2^	440.83	1	440.83	95.83	<0.0001	
*X*_3_^2^	465.40	1	465.40	101.17	<0.0001	
Residual	46.00	10	4.60			
Lack of fit	16.86	5	3.37	0.58	0.58	not significant
Pure error	29.14	5	5.83			
Cor total	1232.41	19				

**Table 3:** ANOVA of the regression model for the response variables (size) and independent variables (*X*_1_, *X*_2_, *X*_3_).

Source	Sum of Squares	Df	Mean Square	F Value	p-Value Prob. > F	
Model	1.111 × 10^5^	9	12,340.24	61.51	<0.0001	significant
*X*_1_	47.39	1	47.39	0.24	0.6374	
*X*_2_	8199.41	1	8199.41	40.87	<0.0001	
*X*_3_	389.21	1	389.21	1.94	0.1938	
*X*_1_*X*_2_	1078.80	1	1078.80	5.38	0.0428	
*X*_1_*X*_3_	605.87	1	605.87	3.02	0.1129	
*X*_2_*X*_3_	731.91	1	731.91	3.65	0.0852	
*X*_1_^2^	65,823.24	1	65,823.24	328.11	<0.0001	
*X*_2_^2^	35557.22	1	35,557.22	177.24	<0.0001	
*X*_2_^3^	15,626.98	1	15,626.98	77.90	<0.0001	
Residual	2006.12	10	200.61			
Lack of fit	1146.01	5	229.20	1.33	0.3803	Not significant
Pure error	860.11	5	172.02			
Cor total	1.131 × 10^5^	19				

**Table 4:** Comparing the predicted values and actual values.

Index	Predicted Value	Actual Value	Deviation%
EE (%)	87.56	89.73 ± 3.45	2.47
Size (nm)	129.04	128.01 ± 5.91	0.79

**Table 5:** The pharmacokinetic parameters of GB following intravenous administration to mice (*n* = 6).

Tissue	Formulation	*AUC*_0__→__∞_ (μg min mL^−1^)	MRT (min)	T_max_ (min)	C_max_ (μg mL^−1^)	CL (L/min/k)	r_e_	DTI
Plasma	GG-inj	349.59	61.25	5	5.56	0.0194		
	GG-LP	991.58	121.69	5	14.84 ^a,c^	0.0066		
	GGB-LP	1256.81	152.17	5	15.24 ^a,b^	0.0052		
Brain	GG-inj	41.65	62.49	10	0.99	0.1562		
	GG-LP	136.85	105.08	15	2.60 ^a,c^	0.0488	3.29	1.16
	GGB-LP	272.12	134.95	15	3.39 ^a,b^	0.0239	6.53	1.82
Lung	GG-inj	289.67	117.20	5	4.53	0.0224		
	GG-LP	308.80	66.25	5	6.20	0.0210	1.07	0.38
	GGB-LP	387.97	110.62	5	5.56	0.0167	1.34	0.37
Heart	GG-inj	203.30	93.96	5	3.82	0.0319		
	GG-LP	283.04	68.34	5	5.07	0.0229	1.39	0.49
	GGB-LP	259.79	85.81	5	4.07	0.0250	1.28	0.36
Liver	GG-inj	181.94	129.53	5	3.06	0.0357		
	GG-LP	303.15	66.19	5	6.09	0.0214	1.67	0.59
	GGB-LP	269.34	80.08	5	4.59	0.0241	1.48	0.41
Spleen	GG-inj	242.15	94.53	5	4.12	0.0268		
	GG-LP	302.11	70.30	5	4.79	0.0215	1.25	0.44
	GGB-LP	261.93	75.79	5	4.28	0.0248	1.08	0.30
Kidney	GG-inj	368.58	67.36	5	6.23	0.0176		
	GG-LP	319.93	72.15	5	5.48	0.0203	0.87	0.31
	GGB-LP	287.46	106.81	5	4.36	0.0226	0.78	0.22

DTI: drug targeting index. * The concentration in plasma is given as μg mL^−1^; ^a^ *p* < 0.05 compared with GG-inj; ^b^ *p* < 0.05 compared with GG-LP; ^c^ *p* < 0.05 compared with GGB-LP.

**Table 6:** Factors and responses in response surface-central composite design.

Independent Variables	Levels				
	−1.682	−1	0	+1	1.682
*X*_1_	6	7.22	9	10.78	12
*X*_2_	5	5.81	7	8.19	9
*X*_3_	10	13.04	17.50	21.96	25
Y_1_ = EE (%)	Maximize				
Y_2_ = particile size (nm)	Minimize				
